# Low cost maize stover biochar as an alternative to inorganic fertilizer for improvement of soil chemical properties, growth and yield of tomatoes on degraded soil of Northern Uganda

**DOI:** 10.1186/s12870-023-04468-5

**Published:** 2023-10-07

**Authors:** Cosmas Wacal, Daniel Basalirwa, John Byalebeka, Mitsuri Tsubo, Eiji Nishihara

**Affiliations:** 1 Department of Crop and Animal Production, Faculty of Agriculture and Environmental Sciences, Mountains of the Moon University, P.O. Box 837, Fort Portal, Uganda; 2 Department of Agriculture and Natural Resources, Faculty of Agriculture, Uganda Martyrs University, P.O. Box 5498, Kampala, Uganda; 3 Department of Soil and Environment, Swedish University of Agricultural Sciences, Box 7014, 750 07 Uppsala, Sweden; 4 Arid Land Research Center, Tottori University, 1390 Hamasaka, TottoriTottori, 680-0001 Japan; 5 Faculty of Agriculture, Tottori University, 4-101 Koyama Minami, Tottori, 680-8553 Japan

**Keywords:** Soil fertility, Maize stover biochar, Tomatoes, Growth, Soil chemical properties, Yield, Profitability

## Abstract

**Background:**

Soil fertility decline due to nutrient mining coupled with low inorganic fertilizer usage is a major cause of low crop yields across sub-Saharan Africa. Recently, biochar potential to improve soil fertility has gained significant attention but there are limited studies on the use of biochar as an alternative to inorganic fertilizers. In this study, we determined the effect of maize stover biochar without inorganic fertilizers on soil chemical properties, growth and yield of tomatoes (*Solanum lycopersicum* L.). A field experiment was conducted in 2022 for two consecutive seasons in Northern Uganda. The experiment included five treatments; inorganic fertilizer (control), biochar applied at rates of 3.5, 6.9, 13.8 and 27.6 t ha^−1^.

**Results:**

In this study, maize stover biochar improved all the soil chemical properties. Compared to the control, pH significantly increased by 27% in the 27.6 t ha^−1^ while total N increased by 35.6% in the 13.8 t ha^−1^. Although P was significantly low in the 3.5 t ha^−1^, 6.9 t ha^−1^ and 13.8 t ha^−1^, it increased by 3.9% in the 27.6 t ha^−1^. Exchangeable K was significantly increased by 42.7% and 56.7% in the 13.8 t ha^−1^ and 27.6 t ha^−1^ respectively. Exchangeable Ca and Mg were also higher in the biochar treatment than the control. Results also showed that plant height, shoot weight, and all yield parameters were significantly higher in the inorganic fertilizer treatment than in the 3.5, 6.9, and 13.8 t ha^−1^ treatments. Interestingly, maize stover biochar at 27. 6 t ha^−1^ increased fruit yield by 16.1% compared to the control suggesting it could be used as an alternative to inorganic fertilizer.

**Conclusions:**

Maize stover biochar applied at 27.6 t ha^−1^ improved soil chemical properties especially pH, N, P and K promoting growth and yield of tomatoes. Therefore, maize stover biochar could be recommended as an alternative to expensive inorganic fertilizers for tomato production in Northern Uganda.

## Introduction

Soil fertility in sub Saharan Africa has continued to decline mainly due to nutrient mining [1], removing more nutrients from the soil through crop harvest and soil erosion [2], than is replaced through addition of organic and mineral fertilizers and other recommended management practices [3]. Nutrient mining, a practice used by smallholder farmers to practice continuous cropping without adding organic fertilizers or other organic sources of nutrients throughout cropping season, is a major problem concerning soil productivity deterioration [4, 5]. In Uganda, research shows that nutrient removal in harvested crops are not compensated through crop residue return, organic and inorganic fertilizers resulting in negative nutrient balances especially nitrogen (N), phosphorus (P) and potassium (K) [6]. Particularly, soil nutrient losses are estimated at 38.1 kg ha^−1^ N, 16.5 kg ha^−1^ P_2_O_5_, and 32.2 kg ha^−1^ K_2_O making a total of 86.8 kg ha^−1^ per year [7]. This has contributed to significantly low crop yields thus persistent food insecurity where majority of Uganda's population depend on agriculture for their livelihoods [8].

To increase crop yields, it is highly recommended that farmers adopt the use of inorganic fertilizers and application of organic amendments to restore soil fertility. However, the average application rate of inorganic fertilizer in Sub-Saharan Africa has remained as low as 14 kg ha^−1^ per year, which is quite below the average in South Asia (141 kg ha^−1^), Europe (154 kg ha^−1^), South America (175 kg ha^−1^), and East Asia (302 kg ha^−1^) [9]. In Uganda, only 24% of smallholder farmers use inorganic fertilizer, making the country one of the lowest fertilizer users in Sub Saharan Africa [10]. Moreover, this percentage is still very far below the Abuja Fertilizer Summit Declaration of 2006, which recommends that African Countries must apply at least 50 kg of nutrients per hectare per year by 2015. The major reason for the low usage is because of the expensive nature of imported inorganic fertilizer which is unaffordable to smallholder farmers [11]. Hence, there is an urgent need for alternative organic amendments with the ability to supply crop nutrient demands. Biochar is such an amendment which has gained significant attention because of its potential to improve soil fertility, increasing crop growth and yield on degraded tropical soils as well as contributing to the mitigation of climate change [12, 13].

Biochar is a soil amendment produced from thermal degradation of organic materials such as crop residues (rice husks, coffee husks, maize stover etc.), animal wastes, through pyrolysis and it has potential to increase crop yields [14, 15]. The use of biochar is an effective way of recycling agricultural wastes with the goal of sustainable crop production and restoration of soil fertility, at the same time reducing the overdependence on inorganic fertilizers [16, 17].

Several researches have shown that biochar application increases growth and yield of vegetables, grains, oilseeds and legumes [18–22]. For instance, biochar produced from fecal matter and applied at rates of 0, 10, 20 and 30 t ha^−1^ together with N rates showed positive results in lettuce growth [23]. The authors observed an increase in fresh weight of lettuce at 20 t ha^−1^ of biochar with 50 kg N ha^−1^ attributed to the increase in P, K and Mg uptake. Improvement in nutrient uptake and yield of crops cultivated with biochar is usually attributed to increased soil pH, EC, organic carbon (OC), available N, P and K [24]. There is scarcity on the use of biochar for production of tomatoes in Uganda.

Tomato (*Solanum lycopersicum* L.) is one of the most important crops cultivated in Uganda for its economic, nutritional and health promoting benefits [25, 26]. Commonly, tomato fruits can be consumed as salads in raw form or cooked for making soup. The fruits are rich in vitamins, minerals, fiber, protein, essential amino acids, monounsaturated fatty acids, carotenoids and phytosterols [27–30]. In addition, lycopene is an important carotenoid found in tomatoes which possess antioxidant, antihypolipidemic, anticarcinogenic activities, and prevents cardiovascular diseases, while promoting cognitive function and preventing osteoporosis [31, 32]. Because of these nutritional and health benefits, the demand and consumption of tomatoes is very high. By 2021, its global production was estimated at 189,133,955 tons, harvested from 5,167,388 ha of land [33]. The same statistics estimated Uganda’s production level at 37,654 tons, harvested from 6,262 ha of land by 2021 [33]. However, the yield of tomatoes (5.8 t ha^−1^) is still far below the potential of 15 t ha^−1^ [33–35]. One of the challenges affecting tomato yield is poor soil fertility which requires urgent attention.

There is still lack of information on the effects of biochar on soil chemical properties, tomato growth and fruit yield [36–39]. Guo et al. [40] recently demonstrated that biochar application may facilitate the reduction of inorganic fertilizer input, being a sustainable practice for enhancing tomato plant growth, fruit quality, and yield. Furthermore, a study using rice husk biochar pyrolyzed at 350 °C and applied at varying rates of 0, 2.5, 5.0 and 7.5 t ha^−1^ found that biochar amended soil significantly enhanced tomato plant height, stem girth, leaf area, flowers, and fruit yields [41]. The authors suggested the application of 7.5 t ha^−1^ of rice-husk biochar could be utilized to increase growth and yield of tomatoes because of the improvement in soil pH, exchangeable cations such as Ca and K as well as CEC and available phosphorus (P). Furthermore, adding 5%, 10%, or 15% of biochar in a peat-based growing medium improved tomato plant water-use efficiency and increased tomato fruit dry-weight yield by up to 32% [40]. Moreover, Li et al. [42] conducted a field experiments using 10, 20, 40 and 60 t ha^−1^ of maize stover biochar in Inner Mongolia, China and found that soil electrical conductivity and tomato yield increased with increasing rates of biochar at an optimal rate of 30 t ha^−1^. All these studies indicate biochar has the potential to improve the growth and yield of tomatoes through its effects on soil fertility and plant health. Therefore, its use as a soil amendment in tomato cultivation may be a promising strategy for improving productivity. However, there are no studies conducted in Uganda to ascertain the ability of maize stover biochar to improve soil chemical properties under tomato production.

Therefore, this study was conducted to determine the effect of biochar on soil chemical properties, growth and yield of tomatoes, with the aim of identifying the optimal maize stover biochar rate which can sufficiently provide nutrients for tomato growth in the absence of inorganic fertilizer.

## Materials and methods

### Location and site description

The field experiment was carried out during the first (March–May) and second (June–September) rainfall seasons of 2022, at the University farm of Uganda Martyrs University, Ngetta Campus Lira, 2.31 30° N, 32.92 88° E. The dominant soil at this site was classified as Plinthosol [43].

**Fig. 1:**
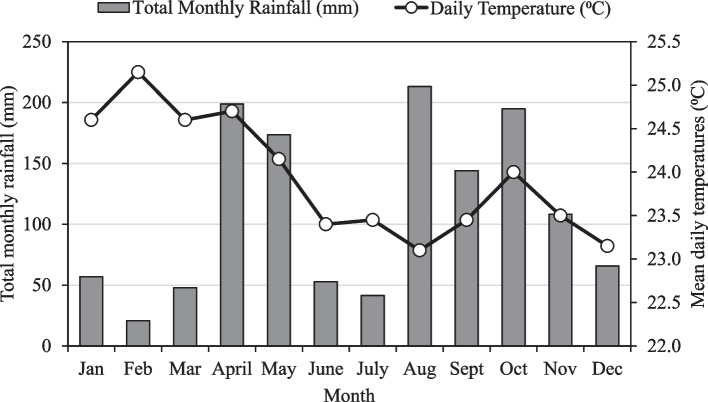
Monthly rainfall and mean daily temperatures received in the study area in 2022

The study area experiences a bimodal rainfall with one peak during April–May and the other in August-October. However, the first season which begins in March and ends in May is characterized by short rainfall while the long rainfall occurs between July and November. The average annual rainfall received in the city was 1318 mm in 2022 (Fig. 1). During the growing seasons, the rainfall peaks occurred in April (198.7 mm), May (173.6 mm) for the first season while highest rainfall was received in August (213.2 mm), followed by October (194.8 mm). Usually, the months of December, January, February, March, June and July receive low rainfall and are considered dry seasons. The average temperatures in the first and second seasons were 24.2^0^C and 23.5^0^C respectively.

The chemical properties of the soil before the experiment used is shown in Table 1 below.

**Table 1:** Soil chemical properties before the experiment

Parameters	Soil before experiment
pH	5.40
EC (dS m^−1^)	0.07
Total carbon (%)	1.40
Nitrogen (%)	0.13
Phosphorus (mg/kg)	6.50
Potassium (m/kg)	140.4
Calcium (mg/kg)	880.0
Magnesium (mg/kg)	253.2
Sand (%)	64
Clay (%)	21
Silt (%)	15
Texture	Sandy clay loam

### Maize stover biochar

#### Field preparation of biochar

**Fig. 2:**
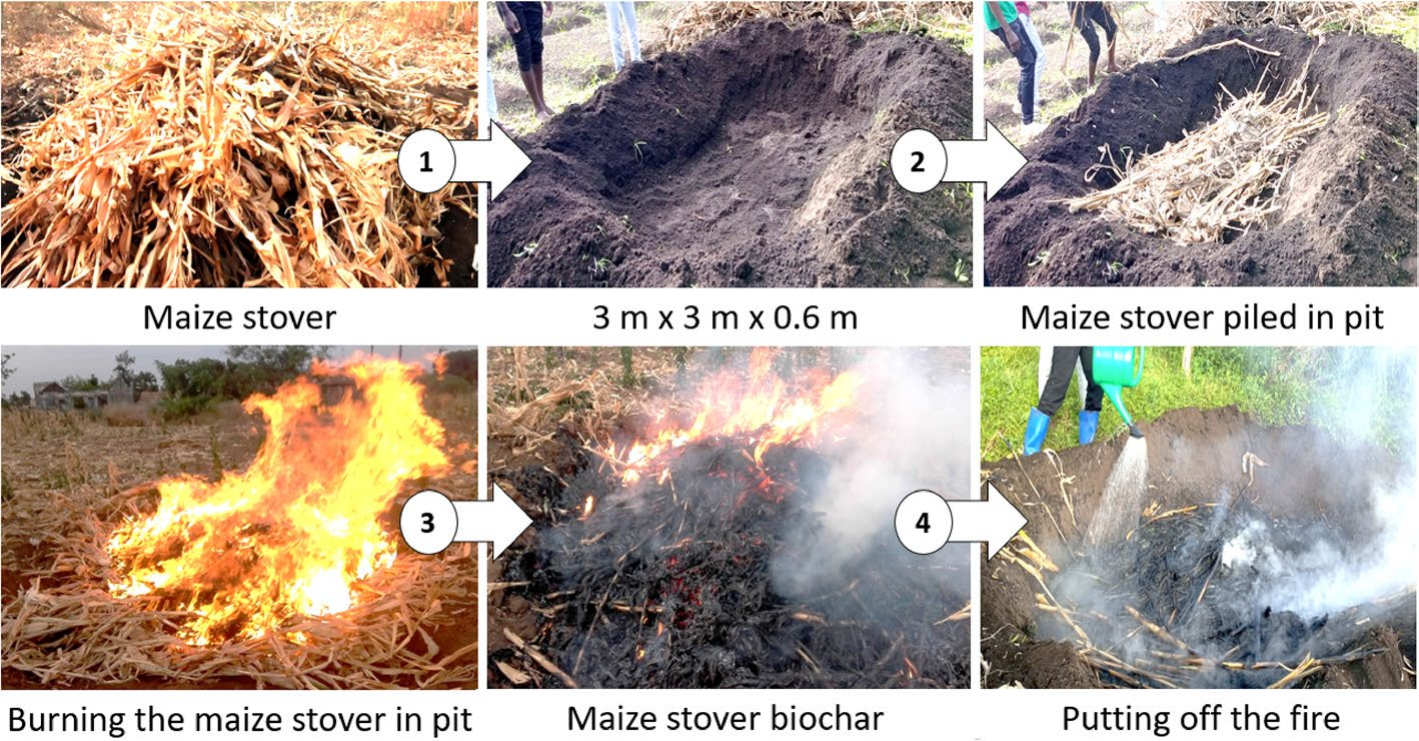
Making biochar in the soil pyrolyzer (open pit method) using maize stover biochar

In this study, we adopted the in-situ field method of biochar preparation in which a soil pyrolyzer (also called a cuboid ditch or open pit method) was employed [44]. This method was chosen because it is cheap (low cost method) and requires low investment in biochar production equipment. Therefore, this fits well within the budget of resource poor smallholder farmers of Uganda [45]. The in-situ biochar preparation was conducted on a maize farmer field situated in Lira City, Uganda where there was abundant air-dried maize stover for the feedstock. Briefly, a soil pyrolyzer (pit) with a dimension of 3 m × 3 m × 0.6 m was made in the field, and a 50 kg pile of air-dried maize stover was loaded into the pyrolyzer (Fig. 2). The maize stover was then set ablaze at both ends of the pyrolyzer and when about 90% of the materials were burnt to red-hot, the fire was quenched by sprinkling water to avoid burning everything in to ash. The resultant materials were then collected and air-dried. This procedure was repeated until the required amount of biochar was obtained. The black material (biochar) pieces were separated from the unburnt materials using hand and a rake. Similar to the open kiln method of biochar preparation, the carbonization of the maize stover is expected to occur beneath the flames under limited oxygen since the flames consume all the feedstock creating a pyrolysis chamber [46]. The maize stover biochar yield was calculated as the weight of biochar in kilograms produced from the 50 kg air-dried feed stock using the formula below.

$$\mathrm{Biochar~yield}\left(\%\right)=\frac{\mathrm{Weight}\;\mathrm{of}\;\mathrm{biochar}\;(\mathrm{kg})}{\mathrm{Weight}\;\mathrm{of}\;\mathrm{maize}\;\mathrm{stover}\;(\mathrm{kg})}\mathrm x100\%$$

### Biochar properties

A sample of 20 g of the maize stover biochar was crushed into powder and passed through a 2-mm sieve prior to determination of its properties. All chemical analysis were performed following the standards described by Okalebo et al*.* [47]. The biochar was analyzed for pH and EC determined from biochar suspension (1:2.5 w/v, biochar: water) mechanically shaken for 1 h and measured with a pH meter (Model pH 700 Meter, Eutech Instruments, Singapore) while EC measured using EC meter (Model Meter HQ40d, Hach Co., UK). The total organic carbon was determined by the potassium dichromate wet acid oxidation method as described by Walkley and Black [48]. Total N was determined by the micro-Kjedahl digestion method as described by Okalebo et al*.* [47]. Exchangeable K, Ca, and Mg were extracted in 1 N ammonium acetate (pH 7.1), and K was analyzed by using a flame photometer (Model Jenway PFP7, Cole-Parmer Co., USA) while Ca and Mg were determined using an atomic absorption spectrophotometer (Model BK-AA320N, Biobase. Co. Ltd, China). Available *P* was determined using the Bray 1 using 0.03 M NH_4_F in 0.025 N HCl and then the P concentration measured by the ammonium molybdate–ascorbic acid method at an absorption wavelength of 880 nm on a spectrophotometer (Model UV-6300 PC, VWR International Co., USA). The ash content was measured by igniting 1.0 g biochar sample at 550 °C for 5 h in a muffle furnace and ash content determined using the following.

$$Ash \left(\%\right)=\frac{Weight~of~ash~(g)}{Weight~of~biochar~(g)} x 100\%$$

### Experimental design

**Fig. 3:**
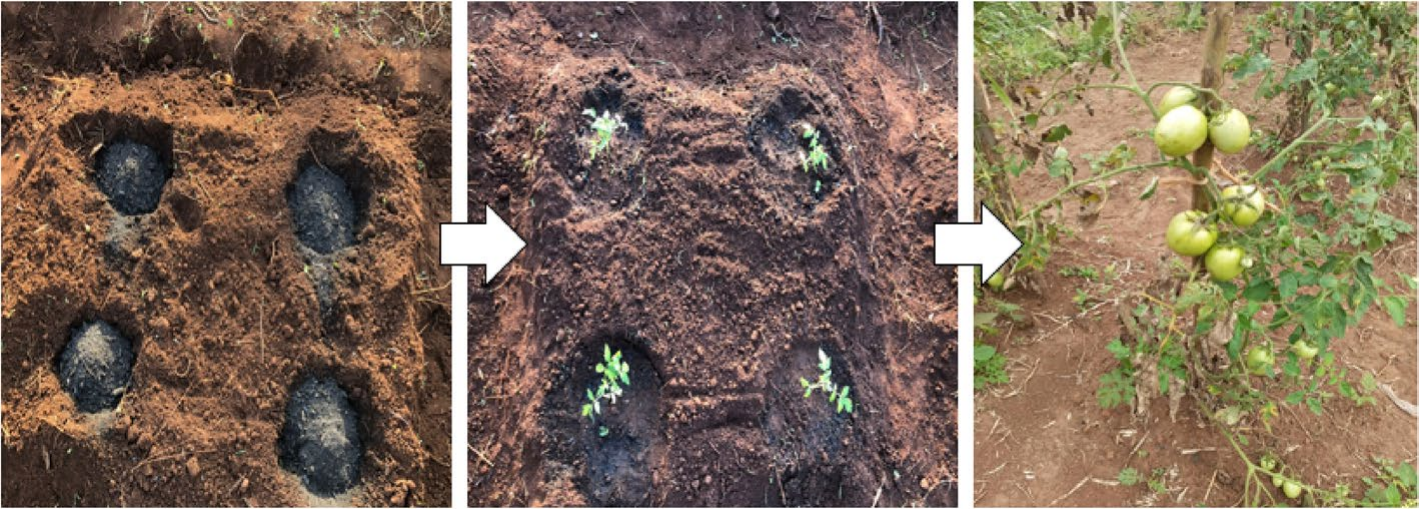
The spot application method of biochar for planting tomatoes

In this study, the experiment was conducted on a field measuring 10 m by 5 m divided into micro plots measuring 1 m by 1 m (1 m^2^). Each micro plot was separated by 1 m as buffer space. Planting holes were made at spacing of 60 cm × 45 cm into which maize stover biochar was applied at varying rates (spot application method) as shown in (Fig. 3). The biochar rates of 0, 3.5, 6.9, 13.8 and 27.6 t ha^−1^ were arranged in completely randomized block design (CRBD) with three replicates. Only the control (0 t ha^−1^) received the recommended basal inorganic fertilizer applied at a rate 185 kg ha^−1^ of diammonium phosphate (N—P_2_O_5_—K_2_O, 18:46:0) which supplied 33 kg N ha^−1^ and 85 kg P_2_O_5_ ha^−1^ while 48 kg N ha^−1^ was obtained from calcium ammonium nitrate (26% N) applied as top dressing. All biochar treatments did not receive inorganic fertilizers since the objective of this study was to evaluate the effectiveness of the biochar in comparison to the inorganic fertilizer.

Tomato variety “Padma F1” was first raised in a nursery bed and after three (3) weeks, were transplanted directly in the holes in which inorganic fertilizer and biochar were incorporated following the spacing of 60 cm × 45 cm (37,037 plants ha^−1^) (Fig. 3). The plants were staked with wood to provide support keeping the foliage off the ground. The plots were kept weed free by regular hand weeding while pests and diseases were managed using recommended chemicals.

The second cropping was conducted on the residual biochar on the same treatments without disturbing the plots during the second season. However, the control received the recommended inorganic fertilizer. All agronomic practices were followed as in the first season.

### Growth and yield analysis

All data on growth and yield analysis were determined at harvest time. Parameters determined included plant height, root weight (g), shoot weight (g), number of fruits per plant, fruit weight per plant (g) and fruit yield (t ha^−1^).

### Soil sampling and chemical analysis

After harvest of the second season, soil samples were collected with an auger to a depth of 15 cm. The soil samples were air-dried, crushed, and passed through a 2-mm sieve and all chemical analyses were performed following standard methods described by Okalebo et al*.* [47]. Soil pH and electrical conductivity was measured in a suspension (1:2.5 w/v soil: water) with a pH meter (Model pH 700 Meter, Eutech Instruments, Singapore) while EC measured using EC meter (Model Meter HQ40d, Hach Co., UK). Soil organic matter was determined by the potassium dichromate wet acid oxidation method as described by Walkley and Black [48]. Briefly, the soil samples were digested with potassium dichromate solution followed by concentrated sulphuric acid before titrating with ferrous ammonium sulphate to yield organic carbon. The amount of organic matter was then calculated by multiplying OC content by 1.724. Total N was determined by the micro-Kjedahl digestion method as described by Bremmer and Mulvaney [49]. Soil exchangeable K, Ca, and Mg were extracted in 1 N ammonium acetate (pH 7.1), and K was analyzed by using a flame photometer (Model; Jenway PFP7, Cole-Parmer Co., USA) while Ca and Mg using an atomic absorption spectrophotometer (Model BK-AA320N, Biobase. Co. Ltd, China). Available *P* was determined using the Bray 1 method in which in which the soil *P* was extracted using 0.03 M NH_4_F in 0.025 N HCl. The *P* concentration in soil samples was then measured by the ammonium molybdate–ascorbic acid method at an absorption wavelength of 880 nm on a spectrophotometer (Model UV-6300 PC, VWR International Co., USA).

### Economic analysis of using maize straw biochar

Economic analysis on the use of maize stover biochar was conducted following the CIMMYT partial budget methodology [50]. Variable costs of biochar and inorganic fertilizer were used for partial budget analysis. Market price of ripe tomatoes at the time of harvest were used for estimating gross income.

Briefly, the Total Revenue (TR) for each treatment was computed by multiplying the market price of tomatoes i.e. Total revenue = Yield × Price for the crop. Total Cost of Production (TCP) was calculated by summing up the costs that vary, including the cost of maize stover biochar and fertilizers in production of tomatoes. The costs of other inputs and production practices such as labor cost for land preparation, planting, weeding and chemical spraying, and harvesting were considered the same for all treatments. The Net profit was calculated by subtracting the total Costs of Production (TCP) from Total Revenue (TR) for each treatment i.e. Net Profit = TR – TCP (Table 2).

**Table 2:** Costs of treatments and production of tomatoes

Season	Costs of Treatment	Costs of Production of Tomatoes (USD^a^ ha^−1^)	Costs of fertilizers (USD ha ^−1^)	Costs of biochar (USD ha^−1^)	Total cost of treatment (USD ha^−1^)
1	Fertilizers	1326.0	396.5	0.0	1722.5
	3.5 t ha^−1^	1326.0	0.0	543.8	1869.8
	6.9 t ha^−1^	1326.0	0.0	1087.6	2413.6
	13.8 t ha^−1^	1326.0	0.0	2175.3	3501.3
	27.6 t ha^−1^	1326.0	0.0	4350.5	5676.5
2	Fertilizers	1326.0	396.5	0.0	1722.5
	3.5 t ha^−1^	1326.0	0.0	0.0	1326.0
	6.9 t ha^−1^	1326.0	0.0	0.0	1326.0
	13.8 t ha^−1^	1326.0	0.0	0.0	1326.0
	27.6 t ha^−1^	1326.0	0.0	0.0	1326.0

^a^1 USD = 3,733 UGX. It is assumed that farmers will obtain maize stover on their farms after harvesting maize and therefore it was not included as a cost

### Statistical analysis

Data were analyzed using a one-way analysis of variance (ANOVA) to examine the effect of biochar on soil chemical properties, growth and yield of tomatoes, using SPSS 20.0 software package (SPSS IncChicago, IL, USA); pairs of means were compared on significant ANOVA tests using Tukey’s honestly significant difference (HSD) test (*p* < 0.05). Unless otherwise noted, differences were considered significant at *p* < 0.05. Results are presented as the mean ± SE (standard errors) of the three replicates.

## Results

### Maize stover biochar yield and properties

At the end of the pyrolysis, the materials in the pit included unburnt feedstock, the biochar and ash. The result of the maize stover biochar analysis is shown in Table 3. The pyrolysis process yielded 17.3 kg of biochar from the 50 kg of maize stover, thus a biochar yield of 34.6%. The biochar produced contained high ash content of (40%), pH of 9.2 and EC of 3.55 dS m^−1^. The maize stover biochar was found to be rich in organic carbon (15.7%), total N (0.97%), and phosphorus (3.08%). It also had high contents of exchangeable cations K, Ca and Mg.

**Table 3:** Biochar properties produced from the soil pyrolyzer

Parameters	Characteristics
Biochar yield (%)	34.6
Ash (%)	40.0
pH	9.20
EC (dS m^−1^)	3.55
Total carbon (%)	15.7
Total Nitrogen (%)	0.97
Phosphorus (%)	3.08
Potassium (%)	4.6
Calcium (%)	1.6
Magnesium (%)	0.1

### Effect of maize stover biochar on soil chemical properties under tomato production

All soil chemical properties were significantly affected by the treatments (Table 4). Result shows that soil pH was significantly increased by biochar application. Compared to the control (inorganic fertilizer), soil pH increased by 21.5% in the 3.5 t ha^−1^ and there was no significant differences among the biochar treatments although biochar rate of 27.6 t ha^−1^ had a tendency to have higher pH (6.37) than in 3.5 t ha^−1^ (5.9). Results further show that the soil electrical conductivity (EC) was significantly highest in the control (0.584 dsm^−1^) which received inorganic fertilizer whereas all biochar treatments had low EC values without any significance differences among them.

**Table 4:** The effect of maize straw biochar on soil chemical properties under tomato production

							mg kg^−1^	
Biochar rate (t ha^−1^)	pH (1:2.5)	EC (dsm^−1^)	OM (%)	OC (%)	N (%)	C/N	Ca	Mg
0	4.63b	0.584a	3.00a	1.74a	0.15b	12.08ab	566.7b	142.4b
3.5	5.90a	0.120b	3.17a	1.84a	0.20ab	9.46ab	886.7a	259.2a
6.9	5.87a	0.119b	3.42ab	1.98ab	0.22ab	9.22ab	886.7a	286.8a
13.8	6.03a	0.142b	3.43ab	1.99ab	0.23a	8.85a	833.3ab	255.6a
27.6	6.37a	0.153b	3.88b	2.25b	0.15ab	15.75b	873.3a	261.2a
*p*-value	*p* < 0.001	*p* < 0.001	*p* < 0.01	*p* < 0.01	*p* < 0.05	*p* < 0.05	*p* < 0.05	*p* < 0.001

Means followed by the same letters are not significantly different at Tukey *p* < 0.05

Both organic matter and carbon contents were significantly increased with increasing rates of biochar. Compared to the control, organic matter and carbon content significantly increased by 22.8% in the 27.6 t ha^−1^ biochar rate. Biochar rate of 3.5 t ha^−1^ had significantly lower (3.17%) than 27.6 t ha^−1^ (3.88%). Organic carbon content also had a similar trend. On the other hand, there was no significant differences in the C/N ratio among the control, 3.5 t ha^−1^, 6.9 t ha^−1^ and 13.8 t ha^−1^. However, significantly higher C/N ratio (15.75) was observed in the 27.6 t ha^−1^ than control (12.08) indicating an increase in the C/N ratio by 23.3% in the 27.6 t ha^−1^ compared to the control. Furthermore, results indicate that biochar application significantly increased the soil total N. Compared to the control, soil total N was significantly increased by 35.6% in the 13.8 t ha^−1^ whereas no significant differences were observed among the control, 3.5 t ha^−1^, 6.9 t ha^−1^ and 27.6 t ha^−1^. However, the total N tended to increase with increase in biochar rates except in the 27.6 t ha^−1^ where it decreased.

**Fig. 4:**
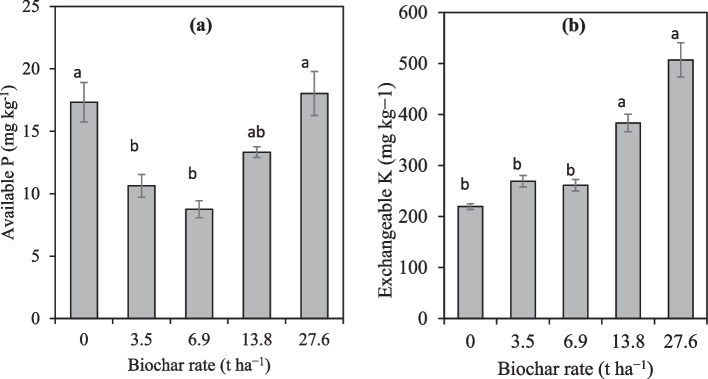
The effect of biochar and fertilizer treatments on the available *P* (**a**) and exchangeable K (**b**) after harvest. Means followed by the same letters are not significantly different at Tukey *p* < 0.05

More still, results show that soil available *P* was significantly decreased by 63%, 97% and 30% in the 3.5 t ha^−1^, 6.9 t ha^−1^ and 13.8 t ha^−1^ respectively compared to the control (Fig. 4a). Interestingly, the soil available *P* was significantly increased by 3.9% in the 27.6 t ha^−1^. In addition, there was a tendency of soil available *P* to increase with increasing rates of biochar. The biochar addition to soil also significantly improved the soil exchangeable cations under tomato production. Compared to the control, exchangeable K was significantly increased by 42.7% and 56.7% in the 13.8 t ha^−1^ and 27.6 t ha^−1^ respectively (Fig. 4b). There were no significant differences among the control, 3.5 and 6.9 t ha^−1^. Compared to the control, soil exchangeable Ca was also significantly increased by 36.1% in both 3.5 t ha^−1^ and 6.9 t ha^−1^ and by 35.1% in the 27.6 t ha^−1^. However, no significant difference was observed between the control and 13.8 t ha^−1^.

Furthermore, results show that exchangeable Mg increased by 45% in the 3.5 t ha^−1^ compared to the control (Table 4). However, the exchangeable Mg content was not significantly different among the biochar rates.

### Effect of maize stover biochar on growth of tomatoes

In the first season, there were no observable differences in the plant heights except it tended to increase non-significantly in the 27.6 t ha^−1^ (Table 5). Root and shoot weights were significantly affected by the biochar rates. Compared to the control, it was observed that root weight was significantly decreased by 127.6% in the 13.8 t ha^−1^ whereas shoot weight decreased by 154.2% for the same treatment compared to control. Interestingly, the 27.6 t ha^−1^ biochar rate increased root weight and shoot weight by 26.0% and 7.6% respectively compared to the control. However, root and shoot weights observed in the 27.6 t ha^−1^ treatment were not significantly different from the control.

**Table 5:** The effect of maize straw biochar on growth parameters of tomatoes

Season	Biochar rate (t ha^−1^)	Plant height (cm)	Root weight (g plant^−1^)	Shoot weight (g plant^−1^)
1	0	87.77a	20.04ab	395.3ab
	3.5	85.64a	13.19bc	189.9bc
	6.9	60.25a	6.81c	67.59c
	13.8	81.58a	8.81c	155.5c
	27.6	95.63a	27.09a	427.6a
	*p*-value	*p* > 0.05	*p* < 0.01	*p* < 0.001
2	0	70.58a	18.66a	91.39ab
	3.5	71.17a	17.63a	70.94bc
	6.9	70.83a	21.07a	51.62c
	13.8	80.31a	27.58a	114.08a
	27.6	78.42a	15.89a	101.78ab
	*p*-value	*p* > 0.05	*p* > 0.05	*p* < 0.001

Means followed by same letters are not significantly different at Tukey *p* < 0.05

**Fig. 5:**
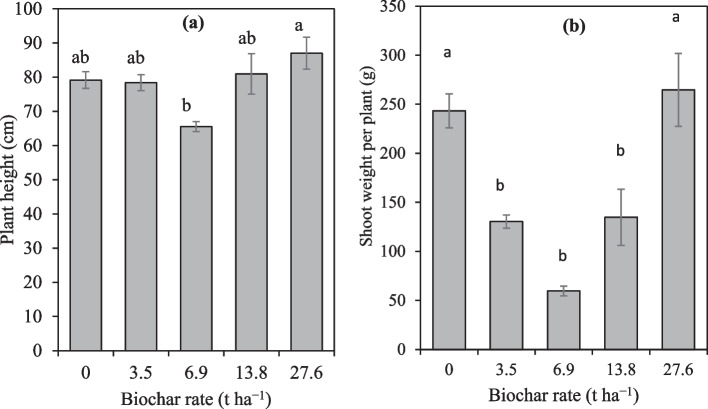
The effect of biochar and fertilizer treatment on the plant height and shoot weight averaged over two seasons. Means followed by same letters are not significantly different at Tukey *p* < 0.05

In the second season, there were no significant differences in the plant height and root weight among the treatments. However, shoot weight was significantly decreased by 77.0% in the 6.9 t ha^−1^ compared to the control. Interestingly, the 13.8 t ha^−1^ significantly increased shoot weight by 19.9% whereas 27.6 t ha^−1^ has increased the same parameter by 10.2% compared to the control. These increments in the 13.8 t ha^−1^ and 27.6 t ha^−1^ were not significantly different from the control. Averaged across the two seasons, there were no significant differences among controls, 3.5, 13.8 and 27.6 t ha^−1^ (Fig. 5a). However 6.9 t ha^−1^ had significantly lower plant height (65.54 cm) than the 27.6 t ha^−1^ (87.02 cm). Overall, the biochar rate of 27.6 t ha^−1^ non-significantly increased plant height by 9.0% compared to control. On the other hand, root weight was non-significantly different among all treatments. With exception of 27.6 t ha^−1^, the biochar rates significantly had lower shoot weight than control (inorganic fertilizer). For instance, the 3.5 t ha^−1^ significantly decreased shoot weight by 86.4% whereas 6.9 t ha^−1^ and 13.8 t ha^−1^ decreased shoot weight by 308.2% and 80.5% respectively, compared to the control (Fig. 5b).

### Effect of maize stover biochar on yield parameters of tomatoes

In the first season, both the control and 27.6 t ha^−1^ had significantly highest number of fruits per plant, fruit weight and yield (Table 6). Number of fruits per plant was significantly decreased by 304.4% and 169.6% in 6.9 t ha^−1^ and 13.8 t ha^−1^ respectively, compared to the control. Similar trends were observed in the fruit weight per plant and fruit yield. In the second season, the number of fruits per plant increased with the increase in biochar rates. Because the control received inorganic fertilizer, it was observed that the low quantities of biochar decreased the number of fruits per plant. Precisely, the number of fruits per plant significantly decreased by 63.0%, 75.4% and 2.7% in 3.5, 6.9 and 13.8 t ha^−1^ respectively whereas 27.6 t ha^−1^ increased the number of fruits per plant by 21.6% compared to the control. Fruit weight per plant and fruit yield followed similar trends.

**Table 6:** The effect of maize straw biochar on yield parameters of tomatoes

Season	Biochar rate (t ha^−1^)	Number of fruits per plant	Fruit weight (g plant^−1^)	Fruit yield (t ha^−1^)
1	0	25.61ab	495.2ab	18.34ab
	3.5	13.00bc	257.1bc	9.52bc
	6.9	6.33c	140.2c	5.19c
	13.8	9.50c	161.6c	5.99c
	27.6	28.50a	570.1a	21.11a
	*p-value*	*p* < 0.01	*p* < 0.01	*p* < 0.01
2	0	8.33ab	197.12ab	7.30ab
	3.5	5.11b	76.02b	2.82b
	6.9	4.75b	78.24b	2.90b
	13.8	8.19ab	167.31ab	6.20ab
	27.6	10.62a	254.91a	9.44a
	*p-value*	*p* < 0.05	*p* < 0.01	*p* < 0.01

Means followed by same letters are not significantly different at Tukey *p* < 0.05

**Fig. 6:**
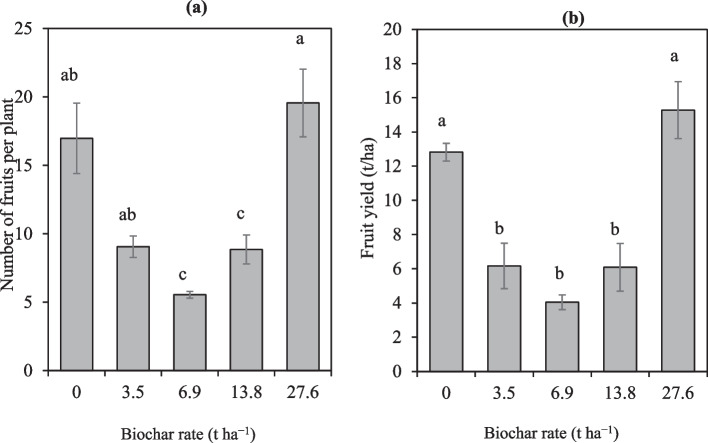
The effect of maize straw biochar on number of fruits per plant and fruit yield. Means followed by the same letters are not significantly different at Tukey *p* < 0.05

Averaged across seasons, the number of fruits per plant was significantly decreased by 206.3% and 91.8% in the 6.9 t ha^−1^ and 13.8 t ha^−1^ respectively, compared to the control (Fig. 6a). Results also show that there was a tendency of biochar to increase the number of fruits per plant with increasing rates. The biochar rate of 27.6 t ha^−1^ significantly increased the number of fruits per plant by 13.2% compared to the control. However, no significant difference was observed between the control (fertilizer) and the 27.6 t ha^−1^.

On the other hand, the biochar rates of 3.5 t ha^−1^, 6.9 t ha^−1^ and 13.8 t ha^−1^ significantly had lower fruit yields than the control with inorganic fertilizer (Fig. 6b). However, at 27.6 t ha^−1^, fruit yield was non-significantly increased by 16.1% compared to the control. Overall, results indicate that biochar rate at 27.6 t ha^−1^ led to significantly high yield comparable to that of the inorganic fertilizer (control).

### Correlation analysis

A Pearson correlation coefficient was computed to determine the relationship between soil chemical properties and growth and yield parameters (Table 7). The results indicate a significant negative relationship between soil pH and electrical conductivity r = 0.89, *p* < 0.01). In addition, soil pH had a significant positive relationship with exchangeable K (*r* = 0.71, *p* < 0.01) Ca (*r* = 0.73 *p* < 0.01) and Mg (r = 0.76, *p* < 0.01). EC had a significant positive relationship with available *P* (*r* = 0.55, *p* < 0.05), whereas OM and OC had significant positive relationship with K (*r* = 0.78, *p* < 0.05). The C/N ratio had a significant negative correlation with total N (*r* = *-*0.65, *p* < 0.01) but positive relationships with *P* (*r* = 0.74, *p* < 0.01), K (*r* = 0.55, *p* < 0.51), shoot weight (*r* = 0.59, *p* < 0.05), number of fruits per plant (*r* = 0.60, *p* < 0.05) and fruit yield (*r* = 0.63, *p* < 0.05). The available *P* on the other had a significant negative relationship with Mg (*r* = -0.52, *p* < 0.05) but positive relationships with shoot weight (r = 0.79, *p* < 0.01), number of fruits per plant (*r* = 0.74, *p* < 0.01), and fruit yield (*r* = 0.75, *p* < 0.01). Exchangeable Ca and Mg had significant positive relationships (*r* = 0.79, *p* < 0.01). However, Mg showed significant a negative relationship with shoot weight (*r* = -0.52, *p* < 0.05). Plant height had significant positive relationships with all yield parameters of tomatoes.

**Table 7:** Correlation analysis among soil chemical, growth and yield parameters

Parameters	pH	EC	OM	OC	CN	N	P	K	Ca	Mg	HP	RW	SW	NFP	FWP	Yield
pH	1															
EC	-0.89^b^	1														
OM	0.69^b^	-0.52^a^	1													
OC	0.69^b^	-0.52^a^	1.00^b^	1												
CN	0.05	0.20	0.51	0.51	1											
N	0.15	-0.33	-0.19	-0.19	-0.65^b^	1										
P	-0.23	0.55^a^	0.11	0.11	0.74^b^	-0.51	1									
K	0.71^b^	-0.42	0.78^b^	0.78^b^	0.55^a^	-0.19	0.46	1								
Ca	0.73^b^	-0.67^b^	0.43	0.43	-0.10	0.04	-0.34	0.44	1							
Mg	0.76^b^	-0.86^b^	0.45	0.45	-0.18	0.22	-0.52^a^	0.39	0.79^b^	1						
HP	0.11	0.12	0.23	0.23	0.36	-0.26	0.52^a^	0.43	-0.22	-0.22	1					
RW	-0.03	0.29	0.29	0.29	0.35	-0.47	0.41	0.27	-0.25	-0.31	0.77^b^	1				
SW	-0.19	0.44	0.15	0.15	0.59^a^	-0.33	0.79^b^	0.34	-0.46	-0.52^a^	0.75^b^	0.71^b^	1			
NFP	-0.11	0.34	0.20	0.20	0.60^a^	-0.21	0.74^b^	0.38	-0.42	-0.46	0.63^a^	0.60^a^	0.96^b^	1		
FWP	-0.16	0.46	0.24	0.24	0.63^a^	-0.39	0.75^b^	0.34	-0.37	-0.47	0.65^b^	0.72^b^	0.94^b^	0.92^b^	1	
Yield	-0.16	0.46	0.24	0.24	0.63^a^	-0.39	0.75^b^	0.34	-0.37	-0.47	0.65^b^	0.72^b^	0.94^b^	0.92^b^	1.00^b^	1

*HP* Plant height, *RW* Root weight, *SW* Shoot weight, *NFP* Number of fruits per plant, *FWP* Fruit weight per plant, *Yield* Fruit yield

^a^ Correlation is significant at the 0.05 level (2-tailed)

^b^ Correlation is significant at the 0.01 level (2-tailed)

### Economic analysis

Biochar application significantly increased the total revenue and net profit in both seasons (Table 8). Averaged across seasons, the treatments 3.5, 6.9, and 13.8 t ha^−1^ produced 4131, 2709, and 4080 USD ha^−1^ respectively indicating that total revenue increased with increase in biochar application. The highest revenue and profits were observed in the 27.6 t ha^−1^ which was not significantly different from the inorganic fertilizer treatment (control).

**Table 8:** Profitability analysis

Season	Treatment (t ha^−1^)	Total Costs of Production (USD ha^−1^)	Total Revenue (USD ha^−1^)	Net Profit (USD)
1	0	1722.5	12,284.0ab	10,561.5a
	3.5	1869.8	6376.5bc	4506.7abc
	6.9	2413.6	3477.2c	1063.5bc
	13.8	3501.3	4009.4c	508.2c
	27.6	5676.5	14,139.8a	8463.2ab
	*p-values*		*p* < *0.01*	*p* < *0.01*
2	0	1722.5	4889.4ab	3166.9ab
	3.5	1326.0	1885.6b	559.6b
	6.9	1326.0	1940.6b	614.5b
	13.8	1326.0	4149.8ab	2823.8ab
	27.6	1326.0	6322.8a	4996.8a
	*p-values*		*p* < *0.01*	*p* < *0.01*
Mean	0	1722.5	8586.7a	6864.2a
	3.5	1597.9	4131.1b	2533.1b
	6.9	1869.8	2708.9b	839.3b
	13.8	2413.6	4079.6b	1666.0b
	27.6	3501.3	10,231.3a	6730.0a
	*p-values*		*p* < *0.001*	*p* < *0.001*

Means followed by same letters are not significantly different at Tukey *p* < 0.05

## Discussion

Biochar has gained global attention because of its multiple benefits, including the amelioration of physicochemical and biological properties, promotion of soil fertility and crop yield, the control of plant diseases, and immobilization of toxic metals and organic pollutants [51–53]. Several positive benefits of biochar on soil properties, growth and yield of cereals, legumes, oilseed crops and vegetables have been reported [13, 21, 22, 54]. However, there are limited studies on the benefits of biochar on smallholder tomato production in Sub Saharan Africa, particularly in Uganda.

The present study demonstrated that maize stover biochar can be prepared using a low cost soil pyrolyzer on a farmer’s field. Results indicated that the maize stover biochar yield was 34.6% which was higher than that reported (13.7%) by Qifa et al. [44] using similar methods but with modification. Qifa et al. [44], employed the same method of digging a pit, burning the maize stover and later covering using soil. However, the present study, used similar pit for burning maize stover but instead of soil cover, the fire was quenched by sprinkling water which prevents burnt materials from turning to ash, thus maintaining relatively higher biochar yield than covering with soil completely which could trap more heat and pyrolysis continues limiting the yield. The biochar yield is also consistent with that obtained by Yang et al. [55], in which maize stover prepared by a slow pyrolysis under oxygen-limited conditions using a modern vertical kiln produced 35% biochar from the feedstock.

Furthermore, the analysis of the biochar composition revealed it is rich in total carbon, total N, P, K, Ca and Mg and with high ash content as well as pH. The high nutrient content could be attributed to the nutrients accumulated in the maize stover biomass during growth and subsequently their concentration in the biochar during the pyrolysis process [56]. This makes the maize stover biochar an important soil amendment for soil fertility since it is rich in plant nutrients required in crop production and thus adoption of biochar could reduce the over dependence on inorganic fertilizers. In addition, the high biochar pH (9.2) in the present study, is an indicator that it could be utilized for raising soil pH on acidic soils of Uganda. This would therefore offer an alternative to the expensive and inaccessible agricultural lime in Uganda.

In this study, the application of maize stover biochar improved chemical properties of degraded soil under tomato production. Our findings corroborate with several pieces of research that biochar application on degraded soil improves chemical properties such as pH, total organic carbon, organic matter, total N, available *P* and exchangeable K [57–59]. For instance, the pH increased by 27% in the 27.6 t ha^−1^ treatment compared to the control (inorganic fertilizer). This result is consistent with Chintala et al. [60] who reported that maize stover biochar had a larger increase in soil pH than switchgrass biochar at all application rates. Furthermore, a study by Mosharrof et al. [61] showed that rice husk biochar significantly increased soil pH, compared to the control. Mosharrof et al. [61] found that soil pH increased by 44.02% with 15 t ha^−1^ of rice husk biochar compared to the control without biochar. These and our results demonstrate that biochar increases soil pH, thus acting as a liming agent to neutralize soil acidity [62, 63]. The initial soil pH before the experiment was 5.40 but with the maize stover biochar application, it increased to an average of 6.0. Usually, when biochar is applied to acidic soils, it raises the pH by neutralizing the excess hydrogen ions and increasing the concentration of hydroxide ions. This leads to a decrease in soil acidity and an increase in soil alkalinity [64]. The alleviation in soil acidity by maize stover biochar is attributed to the high ash content produced during the pyrolysis process, and also the basic cations (Ca, Mg, K) in the biomass that are transformed into oxides, hydroxides and carbonates creating an alkaline condition in the biochar [61]. Overall, the increase in the soil pH is an evidence that maize stover biochar has a high pH buffering capacity and capable of correcting soil acidity in Uganda [65]. Thus, the present study demonstrates that maize stover biochar has potential to alleviate acidity in degraded soils and could therefore offer a cheap alternative option to commercial agricultural lime.

On the other hand, the significantly lower pH of the control (inorganic fertilizer) compared to biochar treatments could be explained by release of hydrogen ions through the nitrification processes of ammonium from the acidifying fertilizers used such as diammonium phosphate (NH_4_)_2_HPO_4_ and calcium ammonium nitrate (Ca)_2_NO_3_). Application of such acidifying fertilizer has become a noticeable cause of soil acidity in Uganda [66]. This study, therefore demonstrates that it is important to apply the right forms of fertilizers while avoiding forms that may cause soil acidity and the adoption of maize stover biochar as a liming material could be highly recommended for smallholder farmers in sub Saharan Africa including Uganda. Furthermore, the biochar treatments improved the soil organic matter as well as the total carbon content compared to the control suggesting the importance of biochar in restoration of organic carbon stocks on degraded soil of Uganda [5]. In this study, we observed an increase in organic matter and carbon content by 22.8% in the 27.6 t ha^−1^ maize stover biochar treatment compared to the control. Our results are consistent with that of Hu et al. [67] who found that adding different proportions of citrus peel biochar and *Cipangopaludina chinensis* shell powder to citrus orchard soil increased soil organic carbon by up to 22.49%. The authors reported that biochar application rates of 1%, 2%, and 4% resulted in gradual increases in soil organic carbon over time. Therefore, our results suggest that maize stover biochar application can effectively enhance the organic carbon content in soil, providing a potential strategy for carbon sequestration and soil health improvement in Sub Sharan Africa including Uganda.

The results also show that total N was significantly higher in the 13.8 t ha^−1^ compared to the control indicating the potential of maize stover biochar to increase soil N availability crops. A study by Amoakwah et al. [68] reported that Maize cob biochar amendment at a rate of 30 t ha^−1^ also improves nitrogen including carbon which is consistent with our results. This improvement in N availability could be linked to the N content in the maize stover biochar itself, the nutrient retention capacity of the biochar. It is known that biochar has the ability to bind nutrients such as nitrogen (N) and phosphorus (P), preventing their leaching from the soil into runoff water [69]. This helps to retain these nutrients in the root zone, making them available for plant uptake. Although the present study did not determine N uptake by tomato plants, it can be hypothesized that increasing N availability in the soil increased its uptake. Nitrogen is an important macronutrient for crop growth and development. It is a structural component of plant as amino acids, chlorophyll, nucleic acids, ATP and phyto-hormones, that are important in biological processes, involving carbon and nitrogen metabolisms, photosynthesis and protein production [70, 71]. Therefore, satisfying the N demand of tomatoes through applying maize stover biochar could improve physiological processes and enhance growth and yield of tomato plant.

However, at higher biochar rate, 27.6 t ha^−1^, the total N slightly reduced, which could be attributed to the immobilization of N and thus its limited availability especially when the C/N becomes high at such rates [72, 73]. This can also be confirmed by the significant negative correlation between C/N ratio and total N (*r* = -0.65, *p* < 0.01) which indicates that as C/N ratio increases, the N availability reduces. Usually, at high C/N ratio, available N (inorganic N) is temporarily locked up by soil microorganisms during the process of decomposition of biochar with high C/N ratio but eventually inorganic N becomes available as decomposition reaches maximum and populations of microbes decreases [74], which negatively affects N release, lowering its availability and consequently uptake. This may also be caused by stimulation of microbial growth by biochar application in soil that later competes with plants for N, further reducing the amount of N available for plant uptake [75]. Interestingly, the high tomato yield at 27.6 t ha^−1^ compared to the control is an indication that there was adequate available N required for maximum productivity of tomatoes. Thus, the N immobilization may not reflect a deficiency of available N as long as a plant meets its N demand. However, further research is required to understand the nutrient uptake of tomatoes under biochar application to confirm this hypothesis.

The present study also showed that soil available *P* significantly increased in the biochar treatment. This result is consistent with a study conducted by Haque et al. [76]. The increase in soil available *P* could be attributed primarily to the increase in soil pH (Table 4). Soil pH which indicates acidity or alkalinity is an important factor regulating nutrient availability [71]. According to Weil and Brady [71], at soil pH less than 5.5 (acidic soil), there is always high sorption of *P* mainly through replacement of hydroxyl ions on crystal lattices, and hydrated Fe and Al by phosphate ions in the soil. To increase availability of *P*, liming is required to decrease Al^3+^ and Fe^3+^ ions and sorption of P on aluminum and iron oxides [77]. Interestingly, biochar application has potential to decrease Al^3+^ ions while increasing available *P* [61]. Additionally, the liming potential of maize stover biochar used in this study positions it as a suitable amendment for increasing availability of *P* on acidic degraded soil of Uganda. Therefore, the observed increases in soil available *P* with biochar at 27. 6 t ha^−1^ is primarily explained by its ability to raise soil pH to arrange favorable for availability of *P*.

Furthermore, we attribute the increase in soil available *P* in the 27.6 t ha^−1^ to the high *P* content contained in the biochar itself (Table 3). According to Kloss et al. [78], during the pyrolysis process of organic materials, macronutrients such as *P* remain in the same amount and thus the concentration of *P* increases the biochar. This could be the reason why maize stover biochar offers more benefits in soil compared to biochar from other feedstocks. For instance, a study by Purakayastha et al. [79] compared biochar prepared from various crop residues and found out that maize stover biochar enhanced the availability of *P* in the soil suggesting it is suitable for enhancing soil fertility and long-term C sequestration.

The present study also showed that maize stover biochar significantly increased soil exchangeable K with highest K contents observed in the 13.8 t ha^−1^ and 27.6 t ha^−1^ compared to the control. Similar results were observed by Zaidun et al. [80] who reported that soil exchangeable K increased by 64.30% and 111.57% by applying 10 t ha^−1^ and 20 t ha^−1^ respectively, of Empty fruit bunch-palm oil mill effluent biochar. In addition, Gautam et al. [81] reported a similar finding in silty loam Nepalese soil, where exchangeable K was increased using biochar at 5 t ha^−1^. The increase in soil exchangeable K is usually attributed to high content of K in the biochar itself and the reduction of K loss through leaching and stimulating the discharge of K from clay minerals [20, 82–84]. In addition, higher ash content can result in higher potassium content in the biochar which consequently increases the soil exchangeable K [21, 22]. Increasing availability of K could have increased its uptake in the plant tissues although this study did not conduct nutrient uptake. It is known that adequate supply of K improves disease and drought resistance in tomatoes by enhancing physiological features related to drought tolerance and improving the quality and resistance of the plants to abiotic stress [85]. For instance, increasing potassium supply and tissue content leads to a reduction in stomatal conductance and increased water content, which helps plants cope with dehydration [86]. Besides, foliar application of potassium chloride under drought stress conditions increases plant growth, yield, and antioxidant enzyme activities, while decreasing the negative effects of drought stress on plant growth [87]. These findings highlight the importance of potassium in plant defense mechanisms and suggest that maintaining optimal potassium levels can enhance plant resistance to various stressors. Therefore it is expected that with adequate soil exchangeable K under biochar treatments, the absorption of K will be enhanced leading to improvement in tomato quality and resistances to abiotic stress.

Apart from the maize stover biochar increasing exchangeable K, exchangeable Ca and Mg were also higher in the biochar treatment than the control which could be attributed to the high contents of Ca and Mg in biochar and its liming ability. Interestingly, the control treatment (inorganic fertilizer only) also exhibited high exchangeable Ca attributed to the calcium nitrate fertilizer applied as top dressing. Ca and Mg play a vital role in tomato growth and development when absorbed. For instance, calcium plays a role in regulating physiological, biochemical, and molecular processes in plants, including enhancing tolerance to abiotic and biotic stresses [88]. Disorders such as Blossom-end rot (BER) which affects tomatoes, caused by calcium (Ca) deficiency in the fruit tissue is a common challenge in tomato production especially in Uganda [89]. Studies have demonstrated that application of Ca can reduce the incidence of BER and improve fruit quality [90]. Therefore, with the adequate supply of exchangeable Ca in the soil as a result of maize stover biochar addition, fruit losses due to blossom end rot disease of tomato could be minimized. On the other hand, Mg plays various roles such as being a central atom of the chlorophyll molecule, carbohydrate partitioning, enzyme activation, and protein synthesis [91]. This implies Mg is important in photosynthesis and therefore adequate supplies of Mg in the soil through maize stover biochar could significantly enhance tomato yield.

The present study demonstrated that due to the improvement of soil chemical properties by maize stover biochar, tomato growth and yield were enhanced. The increase in plant height, number of fruits per plant and fruit yield of tomatoes under biochar treatment is consistent with findings from other authors [92–94]. For instance, Li et al. [95] conducted a study in the semi-arid area of Inner Mongolia, China with maize stover biochar applied at 10, 20, 40 and 60 t ha^−1^ and found that tomato yield was significantly increased at the 40 t ha^−1^ biochar treatment. In the present study, maize stover biochar applied at 27.6 t ha^−1^ had the highest yield compared to the 3.5 t ha^−1^ signifying that tomato plants require relatively larger quantities of biochar capable of supplying adequate nutrients and improvement in soil physico-chemical properties for positive effect. The increase in the fruit yield by the maize stove biochar could be attributed to the increase in shoot weight and plant height that are associated with overall improvement in soil chemical properties especially through soil nutrient availability [96]. This is evidenced by the significantly strong correlation among soil chemical properties and growth and yield properties. For instance, the available *P* had a significant positive relationship with shoot weight (*r* = 0.79, *p* < *0.01*), number of fruits per plant (*r* = 0.74, *p* < *0.01*) and fruit yield (*r* = 0.75, *p* < *0.01*) (Table 8). This is a suggestion that soil available *P* plays significant role in growth and yield of tomatoes. Suthar et al. [97] in their study using bamboo biochar, reported great improvement in growth and fruit quality of tomatoes, attributed increase in the availability of *P*. Phosphorus is an essential element which is a structural component of nucleic acids, sugars and lipids playing a crucial role in various processes such as seed germination, root and shoot development and photosynthesis [98]. Therefore, with adequate supply of P through the maize stover biochar, the growth and yield of tomatoes were enhanced.

Interestingly, the present study has demonstrated that maize stover biochar applied at 27.6 t ha^−1^ without inorganic fertilizer significantly increased plant height and fruit yield comparable with the fertilizer treatment (control). This finding is in agreement with Lakitan et al. [99] who observed that grain yield of rice treated with 1.2 t ha^−1^ biochar was higher than the control without indicating the potential of biochar as an organic fertilizer on degraded soil. Furthermore, Guo et al. [40] in their experiment with varying rates of biochar 0, 30, 50, and 70 t ha^−1^ under drip irrigation and in combination with four N application rates (170, 190, 210, and 250 kg ha^−1^) showed that biochar application at 50 t ha^−1^ while reducing N fertilizer by 24% achieved the greatest tomato yield. This suggested that biochar application in combination with reduced N fertilizer is sufficient to achieve maximum tomato yield. In the present study 27.6 t ha^−1^ produced tomato yield without significant difference from the inorganic fertilizer treatment suggesting maize stover biochar could offer an alternative nutrient source for tomato production in place of inorganic fertilizers. Overall, this study has demonstrated that maize stover biochar rich in nutrients could be a substitute for the expensive inorganic fertilizers for small holder tomato farmers in Uganda [100]. Based on the tomato fruit yield and economic benefits, the 27.6 t ha^−1^ provides the same net income as inorganic fertilizers and thus, is deemed to be the best application rate which can be recommended to tomato farmers in Uganda.

## Conclusion

The present study demonstrated that maize stover biochar without inorganic fertilizers increased the growth and yield of tomatoes. Plant height, shoot weight, fruit number per plant, fruit weight per plant and fruit yield were significantly increased by the 27.6 t ha^−1^. The increase was mainly attributed to the improvement in soil chemical properties especially soil pH, available *P*, and exchangeable K which is a reflection of increase in soil fertility. The study demonstrated that maize stover biochar can become an alternative nutrient source for tomatoes instead of over dependence on expensive inorganic fertilizers. Considering economic benefits, the use of biochar at 27.6 t ha ^−1^ could be adopted in place of inorganic fertilizers and maize stover biochar can be further improved as a promising alternative biochar-based fertilizer. However, a long-term experiment should investigate how soil fertility is maintained over time and understand carbon sequestration as well greenhouse gas mitigation by maize stover biochar.

## Data Availability

All data generated or analyzed during this study are included in this published article.
